# Endocrine Mucin-Producing Sweat Gland Carcinoma: Case Presentation with a Comprehensive Review of the Literature

**DOI:** 10.3390/dermatopathology10030035

**Published:** 2023-09-01

**Authors:** Gerardo Cazzato, Emilio Bellitti, Irma Trilli, Anna Colagrande, Nicoletta Sgarro, Vincenza Sara Scarcella, Teresa Lettini, Giuseppe Ingravallo, Domenico Piscitelli, Leonardo Resta, Lucia Lospalluti

**Affiliations:** 1 Section of Molecular Pathology, Department of Precision and Regenerative Medicine and Ionian Area (DiMePRe-J), University of Bari “Aldo Moro”, 70124 Bari, Italy; anna.colagrande@policlinico.ba.it (A.C.); n.sgarro1@studenti.uniba.it (N.S.); vincenza.scarcella@policlinico.ba.it (V.S.S.); teresa.lettini@uniba.it (T.L.); giuseppe.ingravallo@uniba.it (G.I.); domenico.piscitelli@uniba.it (D.P.); leonardo.resta@uniba.it (L.R.); 2 Anatomic Pathology Unit, “A. Perrino” Hospital, 72100 Brindisi, Italy; emilio.bellitti@asl.brindisi.it; 3 Odontomatostologic Clinic, Department of Innovative Technologies in Medicine and Dentistry, University of Chieti “G. D’Annunzio”, 66100 Chieti, Italy; i.trilli@studenti.uniba.it; 4 Section of Dermatology, Azienda Ospedaliero-Universitaria Policlinico di Bari, 70121 Bari, Italy; lucia.lospalluti@policlinico.ba.it

**Keywords:** EMPSGC, skin neoplasm, mucinous carcinoma, rare neoplasms, PCMC

## Abstract

(1) Background: Endocrine Mucin-Producing Sweat Gland Carcinoma (EMPSGC) is a rare, low-grade, neuroendocrine-differentiated, cutaneous adnexal tumor, officially recognized by the World Health Organization (WHO) Skin Tumors Classification in 2018 as a separate entity and homologue of endocrine ductal carcinoma in situ (eDCIS)/solid papillary carcinoma of the breast. Although it is more frequent in the female sex, between 60 and 70 years old, in the peri-orbital region, EMPSGC has also been described in the male sex, in subjects under 60 and over 80, and in extra-eyelid localizations (cheek, temple, scalp), but also in extra-facial localizations (chest and scrotum). (2) Methods: We present the clinical case of a 71-year-old woman with an undated lesion of the scalp, which presented as a nodule, skin-colored, and 2.5 cm in maximum diameter. We also conduct a comprehensive literature review from 1997 to the end of 2022, consulting PubMed, Scopus, Web of Science (WoS), and Google Scholar using the following keywords: “Endocrine mucin-producing sweat gland carcinoma” and/or “EMPSGC” and/or “skin” and “cutaneous neoplasms”. In addition, we followed the Preferred Reporting Items for Systematic Reviews and Meta-Analyses (PRISMA) guidelines. A total of 253 patients were recorded; 146 were females (57.7%) and 107 were males (42.2%). The vast majority of the lesions were in the eyelids (peri-ocular region), and only a minority of cases involved the cheeks, supra-auricular, retro-auricular, and occipital region, with very rare cases in the scalp, to which the present is also added. (4) Conclusions: The morphological and immunophenotypical features are essential both for the correct diagnosis and to be able to classify this lesion among the corresponding eDCIS/solid papillary carcinoma of the breast, with neuroendocrine differentiation. Recent papers have attempted to shed light on the molecular features of EMPSGC, and much remains to be conducted in the attempt to subtype the molecular profiles of these entities. Future studies with large case series, and especially with molecular biology techniques, will be needed to further add information about EMPSGC and its relationship in the PCMC spectrum.

## 1. Introduction

Endocrine Mucin-Producing Sweat Gland Carcinoma (EMPSGC) is an entity described recently, in 1997, by Flieder A. et al. [1], and is considered a low-grade neuroendocrine neoplasm, homologous to solid papillary/endocrine ductal carcinoma in situ (eDCIS) of the breast in the World Health Organization (WHO) 2018 classification [2]. Epidemiologically, EMPSGC is more common in women than men, and the median age at presentation is 70 years (range 36–84 years) [3]. For a long time, EMPSGC was considered to pertain exclusively to the eyelid region (around the eye, peri-ocular) but, albeit rarely, cases in extra-eyelid localizations have been reported, for example, by Tsai J.H. et al. [4] and Raquena L. and Sangueza O. [3] in their book “Cutaneous Adnexal Neoplasms”; in particular, while the former case was discussed as it was localized in the areolar region [4], the case presented in [3] was localized at the level of a man’s scalp, casting doubt on the possible extra-eyelid localization. Various authors, and even the WHO classification, recognize that EMPSGC may be a precursor of primary mucinous carcinoma of the skin (PCMC), and, therefore, it should be discussed in the differential diagnosis [5]. Histologically, EMPSGCs appear as rather well-circumscribed uni/multi-nodular tumors with solid, cystic, and papillary areas. The nodular areas usually show a solid growth pattern with small, scattered cysts, and, in addition, in some areas, a cribriform arrangement is present, in which the tumor cells grow on a lacelike network and/or a pseudo-rosette pattern [3,4].

From an immunohistochemical point of view, EMPSGC is usually positive for Cytokeratin 7 (CK7), CK8, CK8/18, CKAE1/AE3, CK-CAM5.2, Epithelial Membrane Antigen (EMA), Gross Cystic Disease Fluid Protein-15 (GCDFP-15), WT1, Estrogen Receptor (ER), and Progesterone Receptor (PgR), but it is very important to underline the positivity for Synaptophysin (Syn) and/or Chromogranin A (CgA), Neuron-Specific Enolase (NSE), and CD56 [3,4,5], which are markers of neuroendocrine differentiation.

In this paper, we present a new extra-eyelid localization of EMPSGC in a 71-year-old female, discuss the morphological and immunohistochemical features with a brief mention of the molecular biology information, and, finally, we conduct an extensive and exhaustive review of the world literature with a focus on future perspectives.

## 2. Case Presentation

A 71-year-old woman presented to the Complex Plastic Surgery Unit with an unspecified history of a non-itchy, tan-to-pink nodule, 2.5 cm, on her scalp. The woman denied the presence of any symptoms and reported only high blood pressure that had been treated with Telmisartan for many years without other disease or concomitant treatment. After physical examination, it was decided to remove the nodule which, after fixation in 10% buffered formaldehyde, was sent to the Complex Operative Unit of Pathological Anatomy.

**Figure 1:**
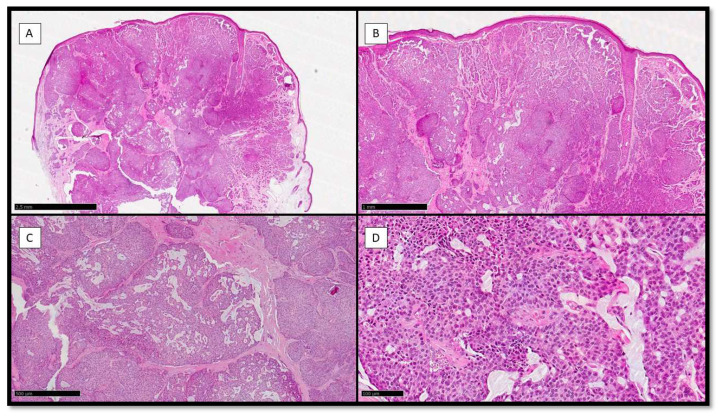
(**A**) Histological preparation for Hematoxylin-Eosin (H&E) showing a polypoid, dome-shaped, well-circumscribed, multinodular neoplasm with solid, cystic, and papillary areas (H&E, Original Magnification 4×). (**B**) Scanning magnification of the previous picture, which shows solid aggregates with a cribriform arrangement (H&E, Original Magnification 10×). (**C**) Histological photomicrograph showing mucin secretion in solid aggregates, with resulting cribriform pattern of growth (H&E, Original Magnification 20×). (**D**) Scanning magnification of the previous picture, showing tumor cells growing on a lacelike network and/or pseudo-rosette pattern with monomorphous, round to oval, and medium size features, and their nuclei with a “salt and pepper” appearance and ample and eosinophilic cytoplasm (H&E, Original Magnification 40×).

Histologically, the neoplasm was multinodular, with solid and sometimes cystic areas (Figure 1A) containing mucin (Figure 1C). Usually, the epithelial aggregates of neoplastic cells were well circumscribed and separated by scant stroma (Figure 1A,B), and there was also the possibility to appreciate areas of cribriform arrangement (Figure 1C) with lacelike network and/or pseudo-rosette pattern of growth (Figure 1D). 

**Figure 2:**
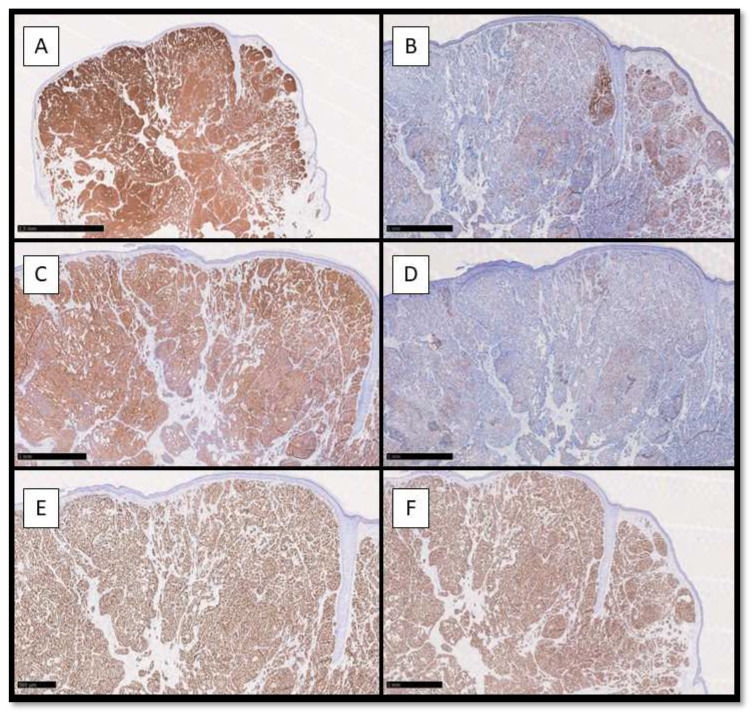
(**A**) Immunohistochemical preparation for anti-CK8/18 antibody: note the diffuse cytoplasmatic positivity of the neoplastic cells constituting EMPSGC (Immunohistochemistry for CK8/18, Original Magnification 4×); (**B**) Immunohistochemical preparation for CK7 antibody: note the partial positivity for CK7 of the neoplastic cells (Immunohistochemistry for CK7, Original Magnification 10×); (**C**) Immunohistochemical photomicrograph showing diffuse positivity for Synaptophysin, marker of endocrine differentiation (Immunohistochemistry for Syn, Original Magnification 10×); (**D**) Photomicrograph showing weak positivity for Chromogranin A (Immunohistochemistry for CgA, Original Magnification 10×). (**E**) Immunohistochemical preparation for Estrogen Receptor: note the diffuse nuclear positivity of the neoplastic cells (Immunohistochemistry for ER, Original Magnification 10×); (**F**) Immunohistochemical preparation for Progesterone Receptor: note that this picture is almost similar to the previous (ER). (Immunohistochemistry for PgR, Original Magnification 10×).

From an immunohistochemical point of view, neoplastic cells were positive for CK8/18 and CK7, with higher positivity for the first one (Figure 2A,B); Synaptophisin was almost positive in a cytoplasmatic pattern, while Chromogranin A was partially positive (Figure 2C,D). Estrogen Receptor (ER) and Progesterone Receptor (PgR) were almost totally positive in the nuclei of the neoplastic cells (Figure 2E,F).

Follow-up data up to 7 months after removal were negative for disease recurrence/metastasis.

In order to recognize and summarize all data present in literature, we also performed a comprehensive review using the following keywords: “Endocrine mucin-producing sweat gland carcinoma” and/or “EMPSGC” and/or “skin” AND “cutaneous neoplasms”, on the PubMed, Scopus, Web of Sciences (WoS), and Google Scholar databases with particular attention to the Preferred Reporting Items for Systematic Reviews and Meta-Analyses (PRISMA) guidelines. Only articles in the English language were selected, and entity discussion articles have been eliminated, favoring case reports and case series, limiting the discussion of other types of information to our ‘discussion’ section.

**Figure 3:**
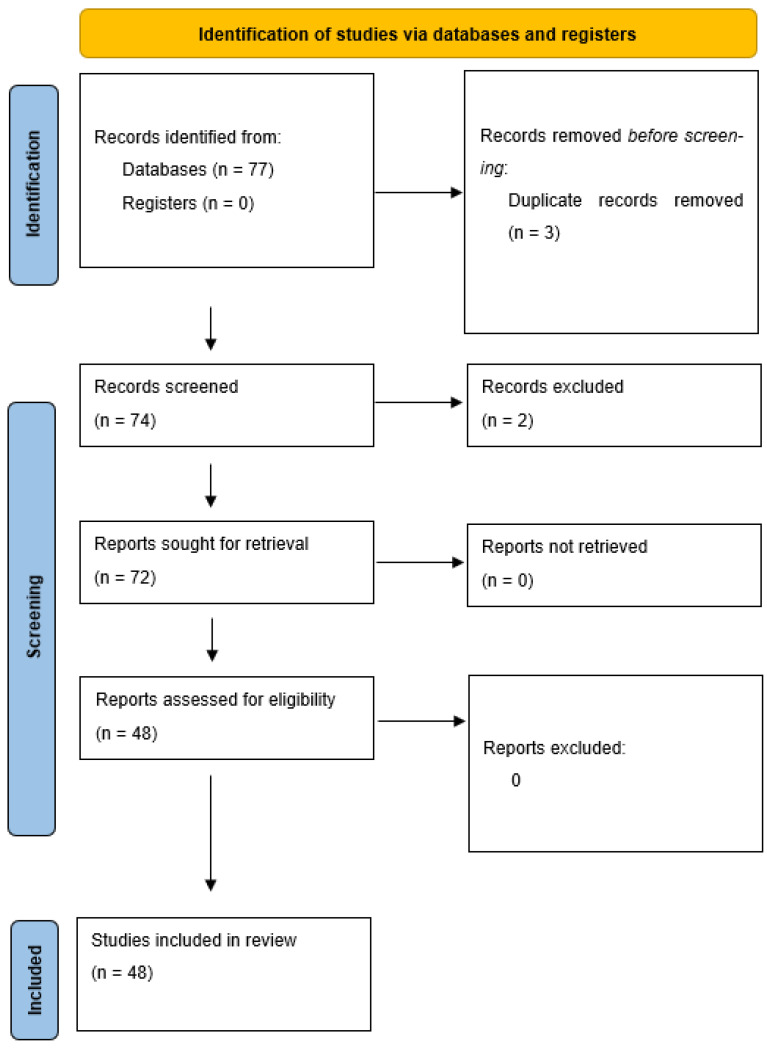
PRISMA guidelines flow-chart followed to perform this review.

Figure 3 presents the features of our review process.

Table 1 summarizes all studies reviewed and analyzed in this paper, with particular attention to year of publication of the papers, number of patients, gender, age of presentation of EMPSGC, localization, and immunohistochemical features. A total of 253 patients were recorded; 146 were females (57.7%), and 107 were males (42.2%). The vast majority of the lesions were in the eyelids (peri-ocular region) and only a minority of cases involved the cheeks, supra-auricular, retro-auricular, and occipital region, with very rare cases in the scalp, to which the present is also added. The average age was reported to be between the sixth and seventh decade, with some cases present before the age of 60 and some present after the age of 80. The most representative immunohistochemistry stains were CK7, CK AE1/AE3, CAM5.2, EMA and, in terms of neuroendocrine markers, Syn, CgA, and NSE, with some cases tested with CD56 (less specific). Furthermore, markers such as GCDFP-15 and Mammoglobin were positive in a good percentage of published cases. With regard to hormone receptors, almost all EMPSGC lesions were positive for at least one of ER, PgR, and AgR. In almost all published cases, immunostaining for p63 and/or SMA was performed to allow the study of the myoepithelium and to understand whether the lesion was in situ or invasive [6,7]. In a paper [8], the diagnostic usefulness of immunohistochemical staining for MYB in 11 cases was reported, while a very recent paper [9] analyzed the use of a new neuroendocrine differentiation marker, Insulinoma-associated protein 1 (INSM1), which was extremely specific and more sensitive than the routinely used neuroendocrine markers, such as Syn and CgA. Finally, in a paper by Mathew et al. [10], immunostaining data were presented on five cases of EMPSGC in which INSM1, AR, BCL2, MUC2, MUC4, RB, Beta-Catenin and MCPyV were tested, of which INSM1 and MUC2 were positive with 4+ intensity, and MUC4 showed 2+/3+ staining mainly at the periphery of the tumor.

Table 2 summarizes the follow-up data of the patients and the clinical outcomes. The vast majority of the lesions were without any recurrence/metastasis, with only a few cases with metastatic or recurrence setting.

## 3. Discussion

EMPSGC represents a very peculiar entity in dermatopathology and ophthalmopathology, and only in the last decade has there been an increasing number of scientific papers studying and shedding light on its histopathological, immunohistochemical, and, moreover, molecular features. Since its initial description [1] up to the latest version of the WHO Skin Tumors [2], the histological and immunophenotypical similarity to its counterpart, referred to as endocrine ductal carcinoma in situ carcinoma (eDCIS) of the breast/solid papillary breast carcinoma, was emphasized, assuming that the very similar embryological nature of the mammary and eccrine gland was the basis for these similarities [3,4,5,6,11,12,13,14,15,16,17,18,19,20,21,22]. 

Epidemiologically, our review of the literature confirms that EMPSGC is more frequent in females, between 60 and 70 years old, with more frequent localization in the peri-ocular region, although there are also reports of other skin sites, such as the cheeks, the scalp, and other very rare localizations such as the skin of the scrotal region, reported in the paper by Shah et al. [54], in which the course of EMPSGC was aggressive, with lymph node, visceral, and bone metastases. A careful analysis of the literature seems to indicate that, although, initially, the published cases of EMPSGC were of the female sex, in the last 10 years, numerous cases have also been published in the male sex, almost in contrast to previous years.

Clinically, it is important to emphasize that EMPSGC does not have a clear profile that allows it to be easily recognized, so much so that, in the papers in the literature, the main suspected clinical diagnoses range from basal cell carcinoma (BCC) to squamous cell carcinoma (SCC), but also Merkel cell carcinoma (MCC) or an innocent epidermal inclusion cyst, hidroadenoma, chalazion, or dermatofibroma (DF). In this regard, Hasegawa-Murakami et al. [35] analyzed the dermoscopic pattern of the case presented in their paper, pointing out that the lesion presented an aggregation of pink to reddish globules (pink ovoid nests), with each globule separated by white to pink meshes of bands (pink network). Furthermore, the red/blue globules were seen in pink ovoid nests of the tumor and, also, some very fine linear–irregular disrupted vessels were recognized. Usually, EMPSGC presents such as a slowly growing skin-colored nodule that can be cystic, multiple, or pigmented [3,42].

In any case, it seems plausible that the clinical diagnosis of EMPSGC does not yet have standardized dermoscopic criteria and that histological examination is always mandatory for the diagnosis.

From a histopathological point of view, the morphological features of EMPSGC are well delineated, with many scientific papers having clearly and comprehensively described the peculiarities of this neoplasm, focusing, in particular, on the well-circumscribed growth, uninodular or even multinodular, with the possibility of having a solid, cystic, and papillary component. In various case reports, it is emphasized that it is possible to find scattered small cysts, and also cribriform aspects, in which the cells are arranged in a pseudo-rosette and/or lacelike patterns. Cytologically, the cells constituting the tumor are monomorphic, round to oval, and of medium/small size. The nuclei have fine granular or stippled chromatin imparting a “salt-and-pepper” appearance, and their cytoplasm is large and eosinophilic. In addition, it is important to emphasize the possibility of secreting mucins, both intracellularly and into the extracellular environment, a characteristic that, over time, has suggested a possible placement of EMPSGC in a spectrum of neoplasms that, at the extreme, would have primitive cutaneous mucinous carcinoma (PCMC) [1,3,4,5,6,8,11,12,13,14,15,16,17,18,19,20,21,22,23,24,25,26,27,28,29,30,31,32,33,34,35,36,37,38,39,40,41,42,43,44,45,62,63].

Although morphology is very important, immunohistochemistry plays a paramount role in the correct diagnostic framing of EMPSGC. All papers published so far show that, with exceptions due to pre-analytical variables, EMPSGC expresses with high concordance Cytokeratin AE1/AE3, CAM 5.2, CK7, and EMA, but also GCDFP-15 and E-cadherin, together with at least one neuroendocrine differentiation marker such as Synaptophysin, Chromogranin A, Neuron-specific Enolase, and/or CD56. Furthermore, it is important to emphasize that this entity expresses Estrogen Receptors (ER) and Progesterone Receptors (PgR) in the vast majority of cases [1,3,4,5,6,8,11,12,13,14,15,16,17,18,19,20,21,22,23,24,25,26,27,28,29,30,31,32,33,34,35,36,37,38,39,40,41,42,43,44,45]. Our case presented immunohistochemical expression for CK8/18 and partially for CK7; it also presented diffuse and strong immuno-expression for Syn and, focally, for CgA. Finally, it had nuclear expression for ER and PgR. 

Molecular data concerning EMPSGC are still limited, although more and more evidence has been published in recent years. For example, Murshed et al. [45] performed Next Generation Sequencing (NGS) analysis on their case of a 78-year-old man with an EMPSGC of the right inferior eyelid. The analysis, conducted on genomic DNA of a tumor extracted from formalin-fixed paraffin-embedded (FFPE) tissue, targeting frequently mutated 59 genes (including EGFR, KRAS, ALK, ROS1, BRAF, HRAS, NRAS, NTRK, AKT1, PIK3CA, KIT, and PDGFRA), disclosed no gene mutations or fusions. On two cases of EMPSGC, Cornejo et al. used NGS with a focused panel of 50 frequently altered genes [27]. The genes EGFR, KRAS, and GNAS, which are frequently involved in mucinous neoplasms, were not found to have alterations in that study. AKT1 and PIK3CA gene alterations, which are typically found in papillary carcinomas of the breast, were also not found. In another two cases of EMPSGC, Qin et al. [34] used array comparative genomic hybridization (aCGH), which revealed a 6p11.2 to 6q16.1 deletion in one of the cases. Held et al. [8] performed an MYB antibody staining of 10 cases of EMPSGC, revealing that all of the cases displayed significant nuclear MYB expression; furthermore, the expression of MYB was found negative in primary mucinous cutaneous carcinomas and mucin-rich basal cell carcinomas, and, also, they found that MYB might be a helpful surrogate measure, particularly in EMPSGC cases with low mucin levels. However, the authors underscore that fluorescent in situ hybridization (FISH) testing on each case in that study came out negative for MYB gene translocation, and amplification is preferable.

In the terms of differential diagnosis, EMPSGC should be carefully distinguished from adnexal lesions such as hydroroadenoma, hydrocystoma with papillary ductal hyperplasia, apocrine adenoma, and apocrine adenocarcinoma. The main modality lies in the correct morphologic framing of the listed lesions, with special attention to the potential recognition of a PCM component, in which case it is mandated to define the lesion as mucinous adenocarcinoma of the skin.

Our comprehensive review of the literature confirms the already widely established data regarding the almost non-existent recurrence and/or distant metastasis of EMPSGC, with very few cases of this one. Interestingly, Froehlich et al. [49] reported a case of EMPSGC of the right eyelid in a patient who had not wanted to undergo surgery after an initial diagnosis and who, upon recurrence, developed a metastasis in 2/9 excised intra-parotid lymph nodes. It is reasonable to state that this result was not due to the (in itself low) potential of the neoplasm, but to the failure to undergo surgery for therapeutic purposes. In another paper by Hadi et al. [52], a case is presented of a 66-year-old subject who developed several recurrences of EMPSGC over a period of nine years, with a metastatic lesion on the ipsilateral parotid gland and a rib. This anecdotal case is of extraordinary importance, as the authors emphasize that the histological and immunohistochemical evaluation of EMPSGC must be very detailed and careful, as potential foci of invasion can easily be overlooked, and not correctly framed within the rarer but aggressive PCMC. Precisely in this regard, several papers analyzed in the literature have proposed to always perform an immunolabelling with Smooth Actin muscle (SMA), or p63 p CK5/6, to study the continuity of the myoepithelium, similar to what happens in the evaluation of breast cancer. Although the data presented are in agreement with this solution, in order to understand whether there is reduction/disappearance of the myoepithelium with consequent potential invasiveness of EMPSGC, a paper by Saggini et al. [64] critically addresses the use of IHC markers for the myoepithelium, as they are prone to error. In particular, referring to the breast, the authors caution against defining as ‘invasive’ an EMPSGC that, while losing myoepithelial IHC markers, always grows in an expansive pattern in the absence of clear tongues of infiltration.

More recently, an interesting paper by Parra et al. discussed a novel immunohistochemical marker called Insulinoma-associated protein 1 (INSM1) that is a transcriptional repressor that plays an essential role in neuroendocrine differentiation [9]. The authors demonstrated that its nuclear expression is stronger and more diffuse than traditional neuroendocrine markers, such as Syn and CgA, and its staining is cleaner and more non-specific than the aforementioned markers.

As far as the etiopathogenesis is concerned, in the current state of knowledge, it is not possible to characterize the carcinogenesis exactly, although a paper by Nishimoto et al. [53] reports a very rare case of multiple EMPSGC/MCS in the same 71-year-old patient, who simultaneously developed a primary mucinous carcinoma of the breast and had a history of unspecified carcinoma of the uterus body some 24 years earlier. The authors correctly hypothesize that the multiple occurrence of hormone-responsive carcinomas (estrogen and progesterone) could be the explanation for why EMPSGC occurs more in the female sex, through a genetic alteration of the receptors.

## 4. Conclusions

In conclusion, EMPSGC represent a rare, low-grade cutaneous adnexal tumor, recognized for the first time with such nomenclature and entity specificity in the 2018 WHO classification. Although, in the early years after the initial description by Flieder et al., there had been few cases reported in the literature, mainly on the eyelids of older women, over time, they have also been described in male subjects, in not only extra-eyelid localizations (such as the case we present here) but also extra-facial topography. The morphological and immunophenotypical features are essential both for the correct diagnosis and to be able to classify this lesion among the corresponding eDCIS/solid papillary carcinoma of the breast, with neuroendocrine differentiation. Recent papers have attempted to shed light on the molecular features of EMPSGC, and much remains to be performed in the attempt to subtype the molecular profiles of these entities. Future studies with large case series, and, especially, with molecular biology techniques, will be needed to further add information about EMPSGC and its relationship in the PCMC spectrum.

## Figures and Tables

**Table 1:** Detailed features of the papers constituting this review.

Author(s)	Year	Patient(s) Gender	Age	Localization	IHC Features (Only Positive)
Flieder et al. [1]	1997	2 female	60, 75	2 eyelids	Syn+ CgA+ ER, PgR+
Tannous et al. [5]	2005	1 female	79	lateral right canthus	Syn+ CgA+ ER, PgR+
Zembowicz et al. [6]	2005	12 cases (8 f, 4 m)	70 (48–84)	8 lower eyelids 2 upper eyelids 2 cheek	At least one of: Syn, CgA, EMA, CK7, CK8/18, ER, PgR
Bulliard et al. [11]	2006	1 female	72	right lower eyelid	Syn+ CK+
Emanuel et al. [12]	2007	1 female	61	left lower eyelid	Syn+ CgA+
Mehta et al. [13]	2008	1 female	70	left upper eyelid	Syn+
Chang et al. [14]	2010	1 male	51	eyelid	CgA, Syn+ ER, PgR+ CK7, EMA+
Inozume et al. [15]	2012	1 male	55	upper cheek	Syn+ CgA+ CK8/18 and CK7+
Salim et al. [16]	2012	2 female 1 male	69 and 53 36	eyelids	Syn+ CgA+ CK7, CEA+ ER, PgR+
Koike et al. [17]	2013	1 male	61	left upper eyelid	focal Syn+
Dhaliwal et al. [18]	2013	2 female	61 and 64	eyelids	Syn+ CgA+ CKAE1/AE3, CAM5.2+ CK7+
Hoguet et al. [19]	2013	11 male 5 female	70 (53–87)	9 upper eyelids 6 lower eyelids 1 not available	All (15): LWCK+ CEA+ ER, PgR+ BRST-2+ At least one of: Syn, CgA, CD56, NSE
Tsai et al. [4]	2014	1 male	57	left chest (areolar)	Syn+ CgA+ ER, PgR+ P63+ (myoepithelial cells)
Shimizu et al. [20]	2014	1 female 1 male	72 74	eyelids	Syn+ CgA+ CKAE1/AE3, CAM5.2+ CK7+
Shon et al. [21]	2014	8 female 5 male	61.2 (40–77 years)	eyelids	WT1, CK7, ER, P-CEA and EMA+ Syn, CgA+
Collinson et al. [22]	2015	1 female	78	left upper eyelid	Ber-EP4+ CK7+ EMA+ Syn, CgA focal+ ER, PgR+
Fernandez-Flores et al. [23]	2015	1 male 1 male 1 female	88 76 69	cheek right upper eyelid right upper eyelid	CK8/18+ CK7+ Syn, CgA+ ER, PgR+
Jedrych et al. [24]	2015	1 female	75	left lower eyelid	CK AE1/AE3+ ER, PgR+ GCDFP-15+ CK7+ Syn focal+
Turnbull et al. [25]	2015	1 male 1 male	62 57	left lower eyelid right lower eyelid	Syn, NSE focal+ CK7+ ER, PgR+ (case 1)
Bamberger et al. [26]	2016	1 male	74	left eyelid	CK7+ NSE+ CD57+ Syn, CgA+ ER+
Cornejo et al. [27]	2016	1 female	71	left upper eyelid	CK CAM5.2+ Syn, CgA, NSE+ ER, PgR+ Ber-EP4+ EMA+
Abdulkader et al. [28]	2016	1 female 1 female	83 51	left lower eyelid right upper lid	CK7+ EMA+ GCDFP-15+ ER, PgR+ Syn, CgA+ GATA-3+ Syn+ CgA focal+ ER, PgR+ GATA-3+
Ross et al. [29]	2017	1 female	29	right lateral lid	NSE+
Brett et al. [30]	2017	1 female	73	upper right eyelid	Syn, CgA, NSE, CD57+ CK CAM 5.2, CK7, GCDFP-15+ ER, PgR+ EMA focal+
Scott et al. [31]	2017	1 female	70	eyelid	Syn, CD56+ CK7+ ER, PgR+
Chou et al. [32]	2017	1 male 1 female	87 55	cheek left upper eyelid	ER, PgR+ GATA-3+ WT1+ Syn+ ER, PgR+ GATA-3+ Syn+
Held et al. [8]	2018	7 male 4 female	66 years (range, 61–84 years	5 lower eyelid 1 upper eyelid 2 cheek 1 supra-auricular 1 retro-auricular 1 occipital	Ber-EP4+ At least one of: Syn, CgA+ CK7, CK CAM 5.2+ ER and/or PgR+ MYB+
Charles et al. [33]	2018	1 male	59	eyelid	Syn, CgA+ CK7, CK CAM 5.2+ ER, PgR+
Qin et al. [34]	2018	8 female 3 male	66 years (56–83)	eyelids and cantus	Syn, CgA+ CK7, CK CAM 5.2+ ER, PgR+
Hasegawa-Murakami et al. [35]	2018	1 male	78	right temple	focal Syn, CgA+ CK7+, CK CAM5.2+ NSE+ ER, PgR+
Nair et al. [36]	2018	1 male	60	left upper lid	CK7, CK8+ ER, PgR+ Syn+ GCDFP-15+ NSE+
Navrazhina et al. [37]	2018	1 female	81	scalp	Syn+ focal CK7+
Kawasaki et al. [38]	2018	1 male	51	eyelid	Syn, CgA+ CK, ER, PgR+
Chen et al. [39]	2018	1 male 1 male	55 87	left cheek left cheek	CK7, GATA3+ Syn, p63, ER, PgR focal+ CK7, GATA3+ ER, PgR+ Syn, CgA focal+
Mulay et al. [40]	2019	7 female 3 male	55–82 (average, 68.7)	eyelids	ER, PgR+ Pan-CK+ EMA, GCDFP-15+ NSE, CgA focal+
Meltzer et al. [41]	2019	1 female	64	left lower eyelid	Syn, CgA+ CK7+
Ansari et al. [42]	2019	7 female 2 male	50–86 (limits)	8 eyelids 1 scalp	At least of: Syn, CgA, NSE+ CK-7, EMA+ RCC+
Nathan et al. [43]	2020	1 female	74	right tragus	Syn, CgA+ CK7+ ER, AgR+
Nasser et al. [44]	2020	1 male 1 female	72 77	left lower eyelids	Ber-EP4+ ER+ CgA+
Murshed et al. [45]	2020	1 male	78	eyelid	CK7, CK8/18+ Syn+ ER, PgR+ GCFDP-15+ EMA+
Bakrin et al. [46]	2020	1 male	59	eyelid	CK7, EMA+ GCDFP-15, Mammoglobin+ ER, PgR+ Syn, CgA+ Syn, CgA+
Shah et al. [47]	2020	1 male	70	eyelid	CK7+ ER+ GATA-3+ Syn+
Agni et al. [48]	2020	42 female 21 male	64 (47–87)	62 eyelids 1 temple	ER, PgR+ Syn, CgA+
Froehlich et al. [49]	2020	1 female	71	right eyelid	Syn, CgA+
Nakamura et al. [50]	2020	4 male	70 72 53 66	cheek cheek lower jaw cheek	CK7+ ER, PgR+ Syn+ CK AE1/AE3+
Katsura et al. [51]	2021	1 male	90	cheek	CK7+ ER, AgR+
Hadi et al. [52]	2021	1 male	66	upper eyelid	GATA-3+
Nishimoto et al. [53]	2021	1 female	71	left cheek and upper eyelids right cheek (multiple lesions)	Mammoglobin+ Syn, CgA focal+ CK7+ ER, PgR+
Shah et al. [54]	2021	1 male	60	scrotum	CgA+ CK7+ ER, GATA-3+ CgA, Syn+
Homer et al. [55]	2021	1 male	40	eyelid	Syn, EMA, CK7+ Syn, CgA+ INSM1+
Parra et al. [9]	2021	5 female 3 male	51–84	4 eyelids, 1 cheek 2 eyelids, 1 cheek	GATA-3, ER, PgR+ CK7, Syn, CgA+ ER, PgR, INSM1+ MYB+
Schafer et al. [56]	2022	6 female 2 male	64 mean	7 eyelids 1 cheek	at least one of NM+
Chuang et al. [57]	2022	3 male	68, 52, 54	infraocular	GCDFP-15+ CK7, ER, PgR+ GATA-3+
Ravi et al. [58]	2022	4 male 3 female	76 (range, 59–98)	eyelids	CK7+ Syn, CgA+
Sarangi et al. [59]	2022	1 male	78	preauricular	ER, PgR, AgR+ GCDFP-15+ Mammoglobin+ Syn, CgA+
Shah et al. [60]	2022	1 male	77	upper lid	at least of NM
Wang et al. [61]	2022	1 female	55	eyelid	CK7+
Mathew et al. [10]	2022	15 female 7 male	71.8 (53–88)	eyelids/peri-orbital	INMS1+ Bcl2+ B-cat+ AgR+ RB1+ partial MUC2+ focal MUC4+

*Legend*. IHC: immunohistochemistry; Syn: Synaptophysin; CgA: Chromogranin A; NSE: Neuron Specific Enolase; ER: Estrogen Receptor; PgR: Progesterone Receptor; AgR: Androgen Receptor; GCDFP-15 (BRST-2): Gross cystic disease fluid protein 15; EMA: Epithelial Membrane Antigen; RCC: Renal Cell Carcinoma; CK: Cytokeratin; LH-CK: Low Weight Cytokeratin; CEA: Carcinoembryonic antigen; INSM1: Insulinoma-associated protein 1; MUC2: Mucin 2; MUC4: Mucin 4; RB1: Retinoblastoma 1; NM: Neuroendocrine Markers.

**Table 2:** Summary of the follow-up data and clinical outcomes of the patients included in the review.

Reference(s)	Sample(s) (n)	Recurrence Follow-Up (Months)	Clinical Outcomes (Recurrence/Metastasis) (n)
[1]	2	72	1
[5]	1	24	0
[6]	12	228	0
[11]	1	7	0
[12]	1	36	1
[13]	1	6	0
[14]	1	-	-
[15]	1	2	0
[16]	3	88	0
[17]	1	6	0
[18]	2	31	0
[19]	16	242	2
[4]	1	30	0
[20]	2	-	-
[21]	13	144	0
[22]	1	8	0
[23]	3	-	-
[24]	1	-	-
[25]	2	-	-
[26]	1	12	0
[27]	1	1	0
[28]	2	13	0
[29]	1	-	0
[30]	1	6	0
[31]	1	-	-
[32]	2	14	0
[8]	11	0	0
[33]	1	-	-
[34]	11	-	-
[35]	1	36	0
[36]	1	6	0
[37]	1	-	-
[38]	1	-	-
[39]	2	16	0
[40]	10	6–36	1
[41]	1	6	0
[42]	9	not retrieved	not retrieved
[43]	1	18	0
[44]	2	17/38	0
[45]	1	24	0
[46]	1	3	0
[47]	1	-	0
[48]	63	1–67	9
[49]	1	not retrieved	1
[50]	4	-	0
[51]	1	42	0
[52]	1	11 months (after metastasis)	1
[53]	1	12	0
[54]	1	-	1
[55]	1	8	0
[9]	8	84 (mean)	0
[56]	8	24 (mean)	0
[57]	3	9/21/7	0
[58]	7	not retrieved	0
[59]	1	108	1
[60]	1	11	0
[61]	1	not retrieved	0
[10]	22	43.25 (mean)	0

## Data Availability

Not applicable.
